# Closing the Gap Between Mammalian and Invertebrate Peripheral Nerve Injury: Protocol for a Novel Nerve Repair

**DOI:** 10.2196/18706

**Published:** 2020-08-27

**Authors:** Maxwell Vest, Addison Guida, Cory Colombini, Kristina Cordes, Diana Pena, Marwa Maki, Michael Briones, Sabrina Antonio, Carmen Hollifield, Elli Tian, Lucas James, Christian Borashan, Johnnie Woodson, John Rovig, Hanaa Shihadeh, Alexander Karabachev, John Brosious, Ashley Pistorio

**Affiliations:** 1 Department of Plastic and Reconstructive Surgery University of Nevada Las Vegas Las Vegas, NV United States; 2 Larner College of Medicine University of Vermont Burlington, VT United States

**Keywords:** Wallerian degeneration, auto-fusion, peripheral nerve injury, nerves, surgery, intervention, rat model, nerve repair

## Abstract

**Background:**

Outcomes after peripheral nerve injuries are poor despite current nerve repair techniques. Currently, there is no conclusive evidence that mammalian axons are capable of spontaneous fusion after transection. Notably, certain invertebrate species are able to auto-fuse after transection. Although mammalian axonal auto-fusion has not been observed experimentally, no mammalian study to date has demonstrated regenerating axolemmal membranes contacting intact distal segment axolemmal membranes to determine whether mammalian peripheral nerve axons have the intrinsic mechanisms necessary to auto-fuse after transection.

**Objective:**

This study aims to assess fusion competence between regenerating axons and intact distal segment axons by enhancing axon regeneration, delaying Wallerian degeneration, limiting the immune response, and preventing myelin obstruction.

**Methods:**

This study will use a rat sciatic nerve model to evaluate the effects of a novel peripheral nerve repair protocol on behavioral, electrophysiologic, and morphologic parameters. This protocol consists of a variety of preoperative, intraoperative, and postoperative interventions. Fusion will be assessed with electrophysiological conduction of action potentials across the repaired transection site. Axon-axon contact will be assessed with transmission electron microscopy. Behavioral recovery will be analyzed with the sciatic functional index. A total of 36 rats will be used for this study. The experimental group will use 24 rats and the negative control group will use 12 rats. For both the experimental and negative control groups, there will be both a behavior group and another group that will undergo electrophysiological and morphological analysis. The primary end point will be the presence or absence of action potentials across the lesion site. Secondary end points will include behavioral recovery with the sciatic functional index and morphological analysis of axon-axon contact between regenerating axons and intact distal segment axons.

**Results:**

The author is in the process of grant funding and institutional review board approval as of March 2020. The final follow-up will be completed by December 2021.

**Conclusions:**

In this study, the efficacy of the proposed novel peripheral nerve repair protocol will be evaluated using behavioral and electrophysiologic parameters. The author believes this study will provide information regarding whether spontaneous axon fusion is possible in mammals under the proper conditions. This information could potentially be translated to clinical trials if successful to improve outcomes after peripheral nerve injury.

**International Registered Report Identifier (IRRID):**

PRR1-10.2196/18706

## Introduction

### Background

Peripheral nerve injuries (PNIs) are devastating and life-altering events that affect 20 million Americans per year and result in an annual economic cost of $150 billion [1]. Despite current nerve repair techniques, recovery from PNIs is often prolonged, incomplete, and associated with poor functional outcomes [2]. After primary repair of a peripheral nerve laceration, regenerating axons frequently contact distal nerve segments that have undergone Wallerian degeneration (WD). WD establishes the environment necessary for axon regeneration. Axons regenerate within Schwann cell tubes at approximately 1 mm per day to reach sensory or motor end organs [3].

In certain invertebrate species, WD does not occur for weeks to months after an axon is severed. The regenerating axons frequently make contact with intact distal segment axons and auto-fuse, which restores axon continuity between proximal and distal segments and enables recovery of nerve function within days [4-6]. Auto-fusion occurs with high specificity, as existing evidence suggests that regenerating axons can recognize and fuse with their original distal ends. The mechanisms underlying this specificity are still being investigated, but it is believed that there is overlap with the molecular players involved in auto-fusion. It is critical to note that membrane-bound receptors on the regenerating axolemmal membranes contact their respective ligands bound to phosphatidylserine on the axolemmal membranes of the distal segment axons. Therefore, axolemmal-axolemmal contact is required for fusion to occur [5].

There are numerous differences between the nerve structure of mammals and invertebrates. Many invertebrates have single axons that innervate a distal target, whereas mammals have nerves with highly organized motor and sensory fascicles containing many axons [3,7]. Peripheral nerves in mammals have complicated architecture consisting of multiple compartmentalized layers. Axons are compartmentalized into fascicles, which can be motor, sensory, or mixed fascicles. Mammalian peripheral nerve axons are encased by Schwann cells and produce myelin sheaths in myelinated nerves. In the nematode *Caenorhabditis elegans*, mechanosensory axons are surrounded by glial cells that do not produce myelin [8].

The most notable difference between mammalian and invertebrate species is the time for WD in the distal segment to occur. In mammals, regenerating axons contact distal segment axons that have undergone WD because of the time delay for regenerating axons to outgrow and reach the distal segment. This alone prevents axon auto-fusion in mammals, even if they do possess the intrinsic machinery necessary for fusion. Because of the delayed WD and presence of molecular fusion mechanisms observed in invertebrates, regenerating axons contact intact distal axons, which leads to auto-fusion [5,9].

Notably, in *C elegans*, reduced retraction of the severed axon ends, reduced degeneration of the distal segment, and an increased number of regenerating axons from the proximal segment strongly correlate with the success of axonal auto-fusion. These factors facilitate fusion by providing a reduction in the distance between the proximal and distal ends of the axon, maintaining an intact distal segment, and providing a greater probability of contact between the two segments [9].

In contrast to the heavily myelinated axons produced by Schwann cells in the mammalian peripheral nervous system, invertebrate axons are surrounded by glia that either do not produce myelin, such as in *C elegans* [8]*,* crayfish [10], and leeches [11], or produce a thin layer of myelin, like earthworms [12]. This begs the question as to whether the presence of heavily myelinated axons could be one factor potentially inhibiting axon auto-fusion in mammals, as myelin is a known inhibitor of nerve regeneration [13].

Additionally, invertebrates such as *C elegans* do not have injury-induced immune responses that are present in mammals [6]. Overall, the immune response to PNI in mammals is conducive to successful nerve regeneration [3]. However, in terms of axon auto-fusion seen in certain invertebrates, the immune response is detrimental because WD must be prevented for auto-fusion to occur.

To date, there is no evidence of spontaneous axonal fusion in mammalian axons after transection. Notably, mammalian axonal fusion can be induced after transection by polyethylene glycol (PEG) fusion techniques [4,14]. PEG fusion has been shown to restore axon continuity and prevent WD. PEG fusion works by removing water molecules between two opposing plasmalemmal membranes, which allows the opposing membranes to contact and fuse [15]. However, PEG fusion techniques do not lead to rapid restoration of preinjury function, which can partly be explained by the nonspecific random fusion of axons with mixed motor and sensory end-organ targets [14]. Although there is no evidence of mammalian auto-fusion, no mammalian study has demonstrated axolemmal-axolemmal contact between regenerating peripheral nerve axons and intact distal axons. No study, to our knowledge, has simultaneously attempted to enhance axon regeneration, prevent WD, prevent myelin obstruction, and limit the immune response to establish contact between regenerating axons and intact distal segment axons to assess fusion competence. This is the aim of our novel nerve repair protocol. It is hypothesized that these conditions can be established in a rat model using a combination of pharmacological and surgical interventions aimed to (1) prevent Wallerian degeneration, (2) enhance axon regeneration, (3), prevent myelin obstruction, (4) limit the injury-induced immune response, and (5) establish contact between severed nerve ends.

### Prevent Wallerian Degeneration

Numerous interventions have demonstrated the ability to delay WD in mammals when implemented prior to nerve injury or within a 4- to 6-hour critical window after the time of injury [16]. These interventions include cryotherapy and the administration of ascorbic acid (vitamin C) [6,7,16-19].

Cryotherapy is also neuroprotective, as demonstrated by multiple studies. Within the nervous system, cryotherapy decreases oxygen demand and preserves energy stores. The net effect of cryotherapy after PNI is delayed WD, reduced membrane disruption, and decreased oxidative stress [20,21].

Marzullo et al [22] showed that rat sciatic nerves maintained electrical conduction in vitro for up to 7 days with cooling to 6-9 °C versus 36 hours at 37-38 °C. Sea et al [23] demonstrated a linear relationship between lower temperatures and delayed WD on rat models. Cooling transected nerves to 13 °C, 23 °C, and 32 °C delayed axon fragmentation up to 10, 6, and 3 days, respectively [23].

Ascorbic acid is an antioxidant that has demonstrated neuroprotective effects. Calixto et al [24] demonstrated that oral administration of ascorbic acid delayed WD up to 7 days versus 3 days in wild-type mice in a sciatic nerve transection model. In this study, ascorbic acid was given in drinking water for 10 days before nerve injury. Immunofluorescence and electron microscopy were used to confirm its protective effects on axon degeneration.

### Enhance Axon Regeneration

In order to establish contact between proximal and distal axons before WD in the distal segment, regenerating axons must sprout and advance in a timely manner. The probability of axon fusion increases with a higher number of regenerating axons [9].

The L-type voltage-gated calcium channel antagonist nimodipine has demonstrated efficacy in nerve regeneration in clinical studies. After unilateral recurrent laryngeal nerve (RLN) transection in 19 patients, vocal cord motion recovered 3 times faster compared with historical controls when the RLN was repaired with adjuvant nimodipine administration [25]. Additionally, a pilot study of nimodipine treatment in patients with facial nerve paresis following maxillofacial surgery showed earlier recovery and improved facial nerve function assessed by the House-Brackmann scale after nimodipine treatment. In this study, regeneration times described in the literature were 2 or 3 times longer compared with nimodipine treatment [26].

Cyanocobalamin (vitamin B_12_) is known to aid in the prevention of neuronal breakdown [27]. Vitamin B_12_ plays a role in various cellular processes that allow for preservation of neuronal function [27,28]. It also has the ability to promote regeneration and improve the function of damaged sciatic nerves by upregulating brain-derived neurotrophic factor expression at the mRNA and protein levels [29]. Additionally, Okada et al [30] demonstrated that high-dose vitamin B_12_ improves nerve function after PNI [30,31].

### Prevent Myelin Obstruction

For many years, it has been recognized that myelin sheaths and myelin debris are present at the site of nerve transection [32,33]. More specifically, observations with electron microscopy have made it apparent that transected axonal ends are capped by collapsed myelin sheaths [34,35].

Lysophosphatidylcholine (LPC), an endogenous lysophospholipid, has been used to induce demyelination in order to study methods to enhance or promote remyelination [36-38]. LPC is commonly injected locally within myelin sheaths to induce rapid demyelination within 30 minutes [39]. Epineural injection leads to localized demyelination. From a morphological standpoint, there is no clear damage to surrounding Schwann cells or axons, which also makes it an ideal molecule to study demyelination [39].

### Limit the Injury-Induced Immune Response

Corticosteroid treatment has been shown to inhibit the inflammatory response and reduce the recruitment of macrophages. Steroids such as dexamethasone act by inhibition of phospholipase A_2_, a critical enzyme in the production of inflammatory cytokines [40]. Additionally, steroids are believed to inhibit lipid peroxidation after PNI and consequently enhance recovery [41].

Dexamethasone has anti-inflammatory effects and has been shown to enhance axon regeneration and functional recovery after PNI in multiple studies [29,42,43]. Using a rat sciatic nerve model, Mohammadi et al [43] demonstrated that topical application of dexamethasone to transected nerve ends placed within a silicone tube resulted in enhanced functional recovery. In another study by Sun et al [29] using a rat sciatic nerve injury model, simultaneous administration of dexamethasone and vitamin B_12_ enhanced axon regeneration of myelinated nerve fibers and improved sciatic functional index (SFI) scores and sensory nerve conduction velocity [29].

### Establish Contact Between Severed Nerve Ends

Severed nerve ends must be in proximity for contact between regenerating axons and distal segment axons to occur. This will be performed with microsuture repair with a silicone tube. The proximal and distal transected nerve ends will be inserted into the silicone tube and secured to the tube with microsuture (eg, 10-0 nylon) to guide regenerating axons to the distally transected nerve end. The epineuria of the proximal and distal ends will be sutured directly to the ends of the silicone tube.

Silicone is nonabsorbable and impermeable to large molecules, which is ideal for controlling and manipulating the local microenvironment [7,44]. This controlled microenvironment has been useful to study the effects of growth factors on nerve regeneration by adding various growth factors within the tube between the transected nerve ends. For the purposes of this study, the silicone tube will surround and protect the transected nerve ends to establish a calcium-free microenvironment and minimize calcium-induced neurotoxicity.

Altogether, these medications and interventions will be used in order to prevent WD, enhance axon regeneration, prevent myelin obstruction, and limit the immune response (Table 1).

**Table 1:** Interventions and evidence.

Goal and intervention class		Intervention	Author	Experimental model	Dose or concentration	Route of administration	Main outcome (improvement vs control)
**Prevent AAD^a^ or WD^b^**							
	Antioxidant	Ascorbic acid (vitamin C)	Calixto et al [24]	Mouse sciatic nerve cut	2.5 mg	Oral mixed in water	Immunofluorescent neurofilament staining (WD delayed 7 days vs 3 days)
	Metabolic demand	Cryotherapy	Sea et al [23]	Rat ventral tail nerves cut	13 °C	Local (water-cooled tail cuff)	Electron microscopy (intact distal axon at 10 days vs 3 days)
**Enhance axon regeneration**							
	Calcium-dependent	Nimodipine	Scheller and Scheller [26]	Human peripheral facial nerve paresis	360 mg	Oral	Clinical evaluation (House-Brackmann 150% improvement)
	Antioxidant	Cyanocobalamin (vitamin B_12_)	Sun et al [29]	Rat sciatic nerve	2 mg/kg	Injection to injury site	Sciatic functional index (recovered 48% vs 38% at 28 days vs control)
Prevent myelin obstruction		LPC^c^	Hall [39]	Mouse sciatic nerve	10 mg/mL	Perineural injection	Myelin dissolved within 30 minutes (N/A^d^)
**Limit the immune response**							
	Immunosuppressants	Dexamethasone	Feng and Yuan [42]	Rat sciatic nerve crush	1-2 mg/kg	Intraperitoneal	Gait and histology (improved ~40%)

^a^AAD: acute axon degeneration.

^b^WD: Wallerian degeneration.

^c^LPC: lysophosphatidylcholine.

^d^N/A: not applicable.

## Methods

### Experimental Goals and Design

The goal of this experiment is to determine if spontaneous axon fusion occurs under the proper conditions. The proposed novel nerve repair protocol will aim to promote axolemmal-axolemmal contact between regenerating axons and intact distal segment axons. Preoperative interventions include administration of multiple medications, such as nimodipine, cyanocobalamin, ascorbic acid, and dexamethasone, which have all been shown to delay WD or enhance axon regeneration. Intraoperative interventions include hypotonic calcium-free saline, ethylene glycol tetraacetic acid (EGTA), and application of a silicone tube to establish a favorable calcium-free microenvironment to delay WD and prevent formation of reactive oxygen species. In addition, LPC will be injected into the distal severed nerve end to dissolve the myelin sheaths that prevent axolemmal-axolemmal contact. Postoperatively, the drug cocktail will be continued until the end of the experiment at 42 days to allow for adequate time for regenerating axons to contact the distal segment axons. Further, cryotherapy in the form of ice packs will be applied immediately postoperatively and continued for 42 days to delay WD and increase the likelihood of axolemmal-axolemmal contact. This study will be concluded at 42 days based on previous studies that demonstrated a plateau of SFI recovery after peripheral nerve repairs [14].

### Study End Points and Modalities

Three study end points will be assessed. The primary objective will be to determine whether axon auto-fusion occurred. This will be analyzed by the electrophysiological conduction of compound action potentials (CAP) and compound muscle action potentials (CMAP). As a secondary end point, this study will assess whether axolemmal-axolemmal contact was achieved by viewing transmission electron microscopy (TEM) cross sections and sagittal sections of the distal segments of axons at different time points. Another secondary end point is the assessment of functional recovery using the SFI. SFI is a standardized gait analysis protocol frequently used to assess functional outcomes after sciatic nerve injury [14,45,46].

### Study Groups and Sample Sizes

This experiment will require the use of 36 rats. The negative control group will consist of 12 rats that will undergo sciatic nerve transection and standard microsurgical nerve repair with microsutures. The experimental group will be 24 rats that will undergo standard microsurgical nerve repair of a severed sciatic nerve with the addition of the preoperative, intraoperative, and postoperative interventions outlined in the novel nerve repair protocol. For the experimental group, 4 rats will be assigned to the behavior group for analysis and will undergo SFI testing preoperatively and postoperatively. For the negative control group, 2 rats will be assigned to the behavior group for analysis and will undergo SFI testing preoperatively and postoperatively. This makes for a total of 6 rats needed for behavioral analysis. For the experimental group, 4 rats will be assigned to electrophysiologic and morphologic analysis at each time point by re-exploring nerve repair sites on postoperative day (POD) 3, 7, 14, 21, and 42, for a total of 20 rats. The negative control group will be set up correspondingly for the electrophysiologic and morphologic analysis groups, except half as many rats will be used. This makes for a total of 10 rats needed for the negative control group and 20 rats needed for the experimental group. For the negative controls, more than one rat for each group will be used in case there are any unexpected issues that arise in one rat.

This study will not have a true control group consisting of unoperated rats. This is because this is a pilot study and axon regeneration after sciatic nerve transection has been thoroughly studied. The negative controls and experimental groups will be compared with unoperated animals in studies performed by Mikesh et al [14] and Ghergherehchi et al [45].

Rats in the negative control group will have their sciatic nerves transected and repaired with microsutures. None of the other interventions described in our protocol will be used for the negative control group. The reason for the negative control group is to compare the results of microsuture repair, the current gold standard repair in the absence of a nerve gap, with this novel repair protocol. This study will assess the outcomes of the proposed nerve repair protocol to the current gold standard microsuture repair.

**Figure 1:**
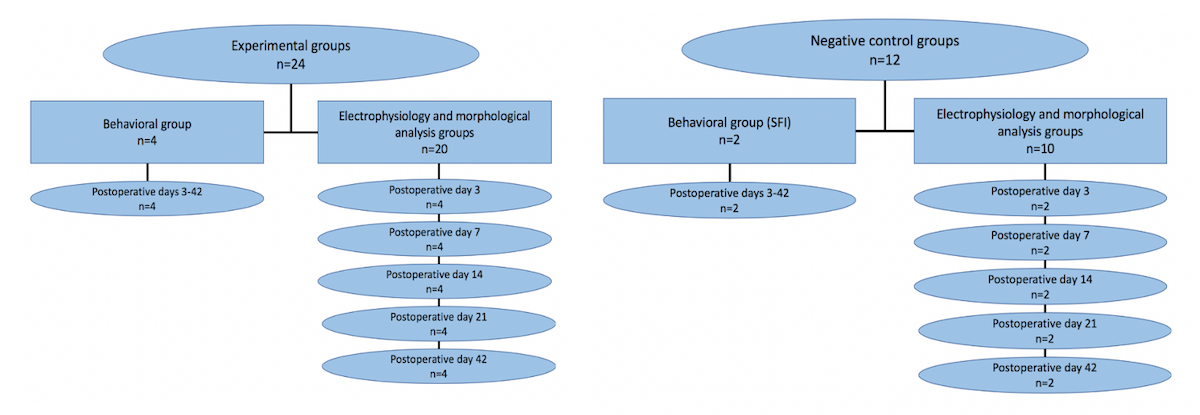
Experimental and negative control groups. The left flowchart represents the behavioral group breakdown and the right flowchart represents the negative control group breakdown. In both experimental and negative control groups, there will be behavioral and electrophysiological/morphological groups. In the electrophysiological/morphological groups, re-exploration and analysis will be performed on postoperative days 3, 7, 14, 21, and 42. SFI: sciatic functional index.

For both the negative control and experimental groups, there will be two separate groups, the behavioral group and the electrophysiology and morphological analysis group. The behavioral groups will be studied using the SFI to determine functional outcomes after the standard nerve repair or the novel nerve repair protocol. The behavioral group will not be euthanized until POD 42. The electrophysiology and morphological analysis groups will be divided into 5 separate groups (POD 3, 7, 14, 21, and 42). Each group will undergo reoperation on their respective postoperative day from the initial operation in order to perform electrophysiological testing. After electrophysiology is performed, the entire sciatic nerve will be harvested for morphological analysis. Once the sciatic nerve is harvested, these rats will be euthanized. A schematic of the protocol methods is summarized in Figure 1.

### Protocol

**Figure 2:**
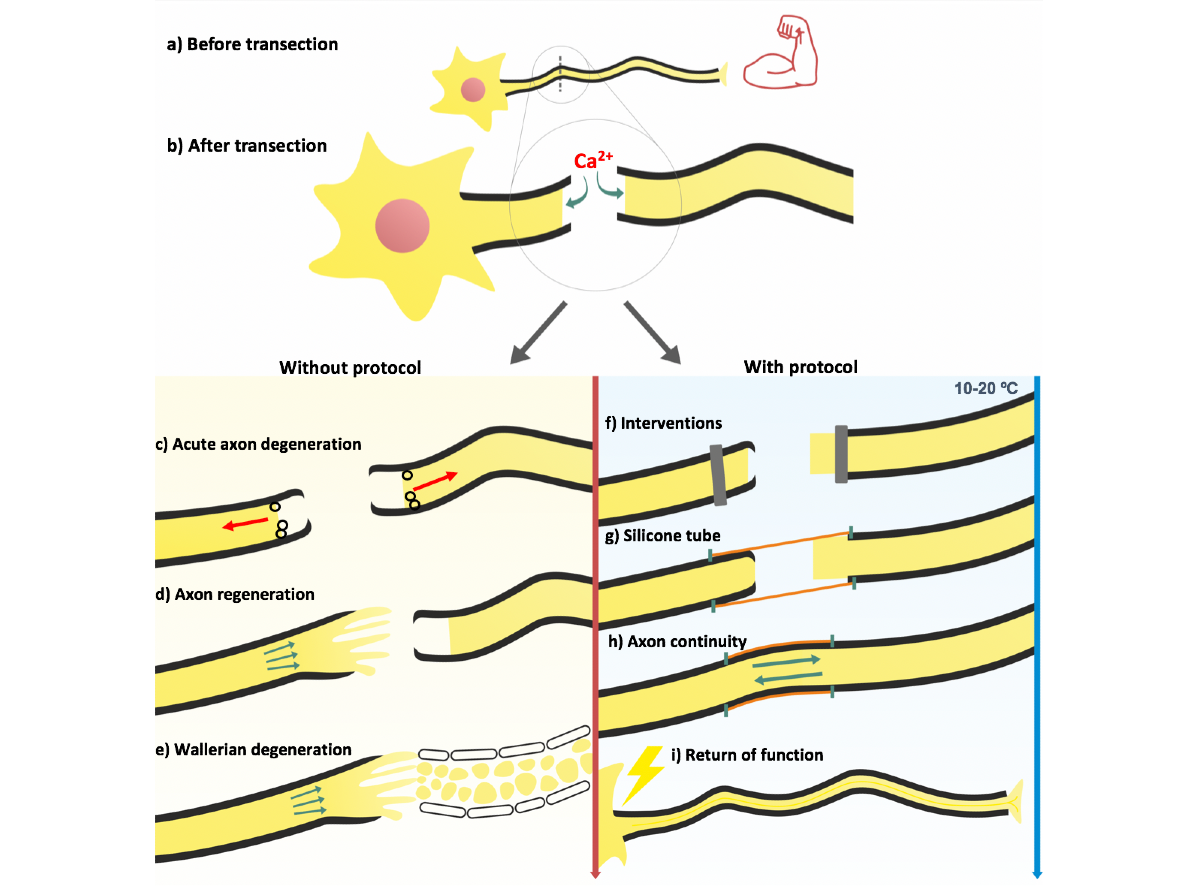
Novel nerve repair protocol. This image compares the usual course of nerve transection (on the left) with the theoretical benefits of the novel nerve repair protocol if fusion occurs (on the right). In (b), note that calcium influx after transection leads to acute axon degeneration, which is noted in (c). Also in (c), membrane seals begin to form (black circles). In (d), axon regeneration begins in the proximal segment. In (e), Wallerian degeneration occurs and regenerating axons regenerate within Schwann cell tubes. In (f), the preoperative interventions have been administered and double approximating clamps (gray vertical lines) are applied to the proximal and distal nerve ends, which will act to seal the axolemmal membranes and prevent further axon degeneration. In (g), the silicone tube is sutured in place after nerve ends have been rinsed with calcium-free saline. If mammals possess the machinery necessary for auto-fusion under the proper conditions, axon continuity could theoretically be restored with return of function, as represented by (h) and (i).

The first part of the protocol will involve preoperative administration of medications, which will start 2 days before the surgical intervention. These medications will continue 42 days after the operation until the conclusion of the study. For the surgical protocol modified from Mikesh et al [14] and Ghergherehchi et al [45], anesthesia will be induced with intraperitoneal injection of ketamine (90 mg/kg) and xylazine (10 mg/kg) [14,45]. The lateral aspect of the left hindlimb will be trimmed and disinfected with 10% povidone-iodine. A 2- to 3-cm incision will be made above the thigh muscle in the left hindlimb of each rat. The biceps femoris muscle will be split in parallel to the muscle fibers with dissection scissors. The sciatic nerve will be trimmed with microdissection scissors to remove any connective tissue. The sciatic nerve will be stimulated to confirm that it can conduct action potentials. The exposed sciatic nerve will be bathed with sterile Plasma-Lyte A (Baxter Healthcare Corp), a calcium-free hypotonic saline solution to prophylactically reduce calcium-induced acute axon degeneration (AAD) that occurs after cut severance. The exposed nerve ends will be bathed in 5-mmol/L EGTA in distilled water to prophylactically chelate extracellular calcium and reduce AAD that occurs after cut severance. Noncrushing double approximating Acland clamps will be applied to proximal and distal ends of the sciatic nerve prior to cut severance to prevent nerve retraction and mechanically create a complete membrane seal to prevent calcium-induced neurotoxicity. Each clamp will be applied 1 cm away from the transection site. Cut severance will be performed by completely transecting the sciatic nerve with a single stroke of dissection scissors to completely sever all the peripheral nerve axons. LPC will be applied to the distal nerve end to dissolve the myelin sheath to allow regenerating axons to contact distal segment axons. Using a micromanipulator, the 1-μm tip of a micropipette will be introduced into the fiber bundle beneath the epineurium and the volume of solution contained within the tip will be injected (about 0.0002 mL) by applying slight positive pressure to the syringe plunger [39]. The silicone tube will be placed to protect and align the severed proximal and distal nerve ends. The ends of the nerves should be in contact within the silicone tube, and 10-0 nylon microsutures will be used to secure the silicone tube to the nerve ends. When suturing the nerve to the silicone tube, the needle will be inserted into the epineural sheath using microneedle holders to avoid axonal damage. Multiple silicone tubes will be available with the proper length and diameter to fit the rat sciatic nerve. The double approximating clamps will then be released. Throughout the procedure, the nerve ends will be moistened with calcium-free solution and EGTA. Skin over the lesion site will be closed with sutures and surgical staples. The behaviorally studied rats will be given a 5 mg/kg subcutaneous injection of ketoprofen after surgery. Immediately after closure, continuous cryotherapy using ice packs will be applied to the left leg distal to the transection site and secured with surgical tape. A diagram of the protocol is demonstrated in Figure 2.

For rats undergoing electrophysiologic and morphological analysis, nerve repair sites will be re-explored on POD 3, 7, 14, 21, and 42. Electrophysiological tests will be performed first to assess nerve continuity (see “Electrophysiological Testing” section below). After electrophysiological recordings are performed, the entire sciatic nerve will be removed. This will be used for morphological analysis by electron microscopy (see “Morphological Analyses of Sciatic Nerves” section below). For the electrophysiologic and morphological analysis groups, postoperative euthanasia will be performed for the POD 3, 7, 14, 21, and 42 experimental group animals after reoperation and harvesting of the sciatic nerve (Textbox 1).

**Novel nerve repair protocol.**

**Preoperative interventions**

- Administer nimodipine 6 mg/kg, gastric gavage once daily (then continue for 44 days)
- Administer cyanocobalamin 1 mg/kg, intraperitoneal injection once daily (then continue for 44 days)
- Administer ascorbic acid 2.5 mg, intraperitoneal injection once daily (then continue for 44 days)
- Administer dexamethasone 1 mg/kg, intraperitoneal injection once daily (then continue for 44 days)

**Intraoperative interventions**

- Expose sciatic nerve with standard microsurgical technique for primary nerve repair
- Confirm sciatic nerve can conduct action potentials by measuring the presence of compound action potentials and compound muscle action potentials
- Bathe exposed nerve in hypotonic calcium-free saline
- Apply noncrushing double approximating Acland clamps to proximal and distal end prior to cut severance
- Apply lysophosphatidylcholine to the distal nerve end using a 1-μm micropipette tip
- Apply a silicone tube and reapproximate nerve ends with 10-0 nylon microsuture
- Release double approximating clips
- Close surgical site

**Postoperative interventions**

- Continue medications (started preoperatively) until postoperative day 42
- Start cryotherapy (10-20 C) and continue until postoperative day 42
- Rats will undergo electrophysiological and morphological analysis by re-exploring nerve repair site on postoperative day 3, 7, 14, 21, and 42
- Behavior analyses by sciatic functional index scores will be performed until postoperative day 42

#### Electrophysiological Testing

Electrophysiological testing will be performed during the initial surgery before and immediately after transection. Electrophysiological testing will be identical to the protocol from Mikesh et al [14] and Ghergherehchi et al [45]. It will also be performed on reoperation for the electrophysiological and morphological analysis study groups on POD 3, 7, 14, 21, and 42 [14,45].

#### Morphological Analyses of Sciatic Nerves

The sciatic nerves will be removed from the electrophysiological and morphological analysis groups on POD 3, 7, 14, 21, and 42 after electrophysiological testing. Morphological analysis will be similar to the protocol from Mikesh et al [14] and Ghergherehchi et al [45]. Nerve fiber diameter and composition will be assessed in the experimental group and compared with the control group. Additionally, qualitative slide analysis will be performed to assess contact of regenerating axons with intact distal axons using TEM.

#### Behavior Analyses

Functional recovery of the sciatic nerve in rats will be assessed using SFI scores identical to the protocols from Mikesh et al [14] and Ghergherehchi et al [45]. SFI testing will be conducted by individuals blind to which procedure the rat has received. Animals will ﬁrst be tested 3 days after surgery, then at POD 7, 14, 21, 28, 35, and 42. A successful recovery is qualified as a score of –59 or better at any time point.

Individual discrepancy can be minimized by using the behavioral analysis protocols outlined by Ghergherehchi et al [45]. This algorithm requires training the behaviorally tested animals for at least one week prior to surgery in order to acclimate the rats to the testing procedures. In practice, the left hindlimb will always be referred to as the experimental (injured) limb. Both hind paws will be dipped in blue (left) or red (right) ink. The rat will then be placed on a wooden board (100 mm in width) lined with white paper strips (100 mm in width) and permitted to run back to their home cage. A trial attempt without any stopping or hesitation during at least three consecutive footprints of both the left and right hindlimb will be considered acceptable as a preprocedure control for later comparison, and the paper strip will be collected for measurements. The normal footprint length, experimental footprint length, normal toe spread, experimental toe spread, normal intermediary toe spread, and experimental intermediary toe spread will be measured in millimeters for all footprints [14,45]. These values will be averaged among the 3 footprints for each limb. Each rat will run 2 trials, and the average will be documented as the SFI score for that rat for that day.

#### Cryotherapy

Immediately after surgical closure, continuous cryotherapy will be applied with ice packs and surgical tape to the left hindlimb distal to the transection site. Skin temperature will be monitored every 6 hours with a digital thermometer. The target temperature range will be between 10 and 20 °C. The ice packs will be continued for 42 days in order to maintain the target skin temperatures. Skin checks will be performed daily to ensure that there are no freeze burns to the skin. The ice will be crushed and wrapped in plastic wrap in order to fit the operated leg.

### Statistical Analysis

As outlined in Mikesh et al [14], Excel (Microsoft Corp) will be used to calculate means, linear regressions, and *t* test comparisons. A 2-tailed Student *t* test will be used to compare mean SFI scores for each treatment group on a given postoperative day. Two-way analyses of variance will be performed to analyze means and standard errors of SFI scores [14,45-47]. CAPs and CMAPs will be recorded as all or none on a binary scale to assess restoration of axon continuity. TEM will be used to demonstrate whether axolemmal-axolemmal contact has been established and will also be recorded as a binary “yes” or “no.”

### Data Monitoring

Dedicated staff will collect and monitor data according to the study protocol and scheduled assessments. Data queries will be addressed as needed. 

## Results

The author is currently in the process of obtaining institutional review board approval. After setup, the study period will take 44 days to complete. The projected completion date, including data analysis and manuscript writing, is December 2021. 

## Discussion

### Contribution to the Literature

The use of PEG fusion has demonstrated that restoring axon continuity and preventing WD alone is not sufficient to regain preinjury function after PNI. This is likely due to the nonspecific fusion of axons that occurs with PEG fusion [4,14]. This begs the question as to whether fusion with high specificity would theoretically restore preinjury function after PNI. More studies will be needed to determine if this is the case. In our study, if fusion does occur, this would reveal that mammals do possess the molecular machinery necessary for auto-fusion and that this mechanism is only expressed under the proper conditions. If auto-fusion is possible, further experiments could be developed to address questions regarding specificity and whether there is mechanistic overlap with fusion, as is demonstrated by *C elegans*. The author believes that these important outstanding questions should be addressed experimentally before determining that mammals do not express the machinery necessary for auto-fusion, especially given the potential benefits to patients that experience PNI. Additionally, if mammalian peripheral axon auto-fusion occurs, further studies could assess if these mechanisms are present in the central nervous system and could be directed to improve outcomes after brain and spinal cord injuries.

### Potential Adverse Outcomes and Preventative Measures

There is always a potential that the protocol could worsen axon regeneration and functional outcomes compared with the standard microsuture nerve repair. One factor that may impair functional recovery is experimentally delaying WD. It is important to note that although preventing WD is necessary for auto-fusion, it is detrimental and inhibitory to axon regeneration. Therefore, if fusion does not occur, axon regeneration may be negatively impacted. This outcome will be monitored with SFI testing in the negative control group, as described earlier.

### Limitations

Because there are multiple mechanisms involved in axon regeneration and WD, the author believes it is important to provide multiple medications and interventions to address the different pathways. This type of study design inherently introduces confounding variables. However, the author believes that addressing each mechanism simultaneously is essential to providing the conditions necessary to achieve the goals outlined by this protocol.

This study does not address nerve gaps. Nerve gaps are common after PNI in humans, as separated nerve ends retract after transection. These are often treated with nerve conduits, allografts, or autografts [48]. This study focuses on sharp nerve lacerations with minimal to no gap between nerve ends. Nerve gaps will need to be addressed in additional future studies.

Data are lacking in higher mammals such as primates and humans for the interventions outlined in this protocol, which is another limitation of this study. The majority of research has been performed on mice, rats, and a few invertebrate species, such as *C elegans*.

### Conclusion

Outcomes after peripheral nerve injuries and nerve repair are poor. More research needs to be done to improve nerve repair protocols. In this study, the efficacy of a proposed novel peripheral nerve repair protocol will be evaluated using behavioral and electrophysiological parameters. The author believes this study will provide information regarding whether spontaneous axon fusion is possible in mammals under the proper conditions. If successful, this information could potentially be translated to clinical trials to improve outcomes after peripheral nerve injury.

## Abbreviations

AAD: acute axon degeneration
CAP: compound action potential
CMAP: compound muscle action potential
EGTA: ethylene glycol tetraacetic acid
LPC: lysophosphatidylcholine
PEG: polyethylene glycol
PNI: peripheral nerve injury
POD: postoperative day
RLN: recurrent laryngeal nerve
SFI: sciatic functional index
TEM: transmission electron microscope
WD: Wallerian degeneration

## References

[1] Taylor CA, Braza D, Rice JB, Dillingham T (2008). The Incidence of Peripheral Nerve Injury in Extremity Trauma. American Journal of Physical Medicine & Rehabilitation.

[2] Ruijs ACJ, Jaquet J, Kalmijn S, Giele H, Hovius SER (2005). Median and ulnar nerve injuries: a meta-analysis of predictors of motor and sensory recovery after modern microsurgical nerve repair. Plast Reconstr Surg.

[3] Mackinnon SE (2015). Nerve Surgery. 1st ed.

[4] Bittner G, Sengelaub D, Trevino R, Peduzzi J, Mikesh M, Ghergherehchi C, Schallert T, Thayer W (2016). The curious ability of polyethylene glycol fusion technologies to restore lost behaviors after nerve severance. J Neurosci Res.

[5] Neumann B, Linton C, Giordano-Santini R, Hilliard MA (2019). Axonal fusion: An alternative and efficient mechanism of nerve repair. Prog Neurobiol.

[6] Teoh J, Wong M, Vijayaraghavan T, Neumann B (2018). Bridging the gap: axonal fusion drives rapid functional recovery of the nervous system. Neural Regen Res.

[7] Lundborg G (2005). Nerve Injury and Repair: Regeneration, Reconstruction, and Cortical Remodeling. 2nd ed.

[8] Oikonomou G, Shaham S (2011). The glia of Caenorhabditis elegans. Glia.

[9] Abay ZC, Wong MY, Teoh J, Vijayaraghavan T, Hilliard MA, Neumann B (2017). Phosphatidylserine save-me signals drive functional recovery of severed axons in Caenorhabditis elegans. Proc Natl Acad Sci USA.

[10] Knowles L (2017). The Evolution of Myelin: Theories and Application to Human Disease. J Evol Med.

[11] Coles JA, Squire LR (2009). Glial cells: invertebrate. Encyclopedia of Neuroscience.

[12] Roots B I, Cardone B, Pereyra P (1991). Isolation and characterization of the myelin-like membranes ensheathing giant axons in the earthworm nerve cord. Ann N Y Acad Sci.

[13] Fawcett J, Schwab M, Montani L, Brazda N, Müller HW (2012). Defeating inhibition of regeneration by scar and myelin components. Handb Clin Neurol.

[14] Mikesh M, Ghergherehchi CL, Hastings RL, Ali A, Rahesh S, Jagannath K, Sengelaub DR, Trevino RC, Jackson DM, Bittner GD (2018). Polyethylene glycol solutions rapidly restore and maintain axonal continuity, neuromuscular structures, and behaviors lost after sciatic nerve transections in female rats. J Neurosci Res.

[15] Donaldson J, Shi R, Borgens R (2002). Polyethylene Glycol Rapidly Restores Physiological Functions in Damaged Sciatic Nerves of Guinea Pigs. Neurosurgery.

[16] Chang B, Quan Q, Lu S, Wang Y, Peng J (2016). Molecular mechanisms in the initiation phase of Wallerian degeneration. Eur J Neurosci.

[17] Hall ED (1987). Intensive anti-oxidant pretreatment retards motor nerve degeneration. Brain Research.

[18] Knöferle J, Koch JC, Ostendorf T, Michel U, Planchamp V, Vutova P, Tönges L, Stadelmann C, Brück W, Bähr M, Lingor P (2010). Mechanisms of acute axonal degeneration in the optic nerve in vivo. Proc Natl Acad Sci U S A.

[19] Nedeljkovic P, Zmijanjac D, Draskovic-Pavlovic B, Vasiljevska M, Vucevic D, Bozic B, Bumbasirevic M (2017). Vitamin B complex treatment improves motor nerve regeneration and recovery of muscle function in a rodent model of peripheral nerve injury. Arch biol sci (Beogr).

[20] Ren X, Orlova EV, Maevsky EI, Bonicalzi V, Canavero S (2016). Brain protection during cephalosomatic anastomosis. Surgery.

[21] Canavero S, Ren X, Kim C, Rosati E (2016). Neurologic foundations of spinal cord fusion (GEMINI). Surgery.

[22] Marzullo TC, Britt JM, Stavisky RC, Bittner GD (2002). Cooling enhances in vitro survival and fusion-repair of severed axons taken from the peripheral and central nervous systems of rats. Neuroscience Letters.

[23] Sea T, Ballinger ML, Bittner GD (1995). Cooling of peripheral myelinated axons retards Wallerian degeneration. Exp Neurol.

[24] Calixto A, Jara JS, Court FA (2012). Diapause formation and downregulation of insulin-like signaling via DAF-16/FOXO delays axonal degeneration and neuronal loss. PLoS Genet.

[25] Mattsson P, Frostell A, Björck G, Persson JKE, Hakim R, Zedenius J, Svensson M (2018). Recovery of Voice After Reconstruction of the Recurrent Laryngeal Nerve and Adjuvant Nimodipine. World J Surg.

[26] Scheller K, Scheller C (2012). Nimodipine promotes regeneration of peripheral facial nerve function after traumatic injury following maxillofacial surgery: an off label pilot-study. J Craniomaxillofac Surg.

[27] Jolivalt CG, Mizisin LM, Nelson A, Cunha JM, Ramos KM, Bonke D, Calcutt NA (2009). B vitamins alleviate indices of neuropathic pain in diabetic rats. Eur J Pharmacol.

[28] Hobbenaghi R, Javanbakht J, Hosseini E, Mohammadi S, Rajabian M, Moayeri P, Aghamohammad Hassan M (2013). Neuropathological and neuroprotective features of vitamin B12 on the dorsal spinal ganglion of rats after the experimental crush of sciatic nerve: an experimental study. Diagn Pathol.

[29] Sun H, Yang T, Li Q, Zhu Z, Wang L, Bai G, Li D, Li Q, Wang W (2012). Dexamethasone and vitamin B(12) synergistically promote peripheral nerve regeneration in rats by upregulating the expression of brain-derived neurotrophic factor. Arch Med Sci.

[30] Okada K, Tanaka H, Temporin K, Okamoto M, Kuroda Y, Moritomo H, Murase T, Yoshikawa H (2010). Methylcobalamin increases Erk1/2 and Akt activities through the methylation cycle and promotes nerve regeneration in a rat sciatic nerve injury model. Exp Neurol.

[31] Altun I, Kurutaş E (2016). Vitamin B complex and vitamin B12 levels after peripheral nerve injury. Neural Regen Res.

[32] Lubińska L (1956). Outflow from cut ends of nerve fibres. Experimental Cell Research.

[33] Freed W, de Medinaceli L, Wyatt R (1985). Promoting functional plasticity in the damaged nervous system. Science.

[34] de Medinaceli L, Freed WJ (1983). Peripheral nerve reconnection: Immediate histologic consequences of distributed mechanical support. Experimental Neurology.

[35] Donat JR, Wiśniewski H M (1973). The spatio-temporal pattern of Wallerian degeneration in mammalian peripheral nerves. Brain Res.

[36] Cole KLH, Early JJ, Lyons DA (2017). Drug discovery for remyelination and treatment of MS. Glia.

[37] Franklin RJM, Gallo V (2014). The translational biology of remyelination: past, present, and future. Glia.

[38] Plemel JR, Liu W, Yong VW (2017). Remyelination therapies: a new direction and challenge in multiple sclerosis. Nat Rev Drug Discov.

[39] Hall SM, Gregson NA (1971). The in vivo and ultrastructural effects of injection of lysophosphatidyl choline into myelinated peripheral nerve fibres of the adult mouse. J Cell Sci.

[40] Keenan GF (1997). Management Of Complications Of Glucocorticoid Therapy. Clinics in Chest Medicine.

[41] Galloway EB, Jensen RL, Dailey AT, Thompson BG, Shelton C (2000). Role of topical steroids in reducing dysfunction after nerve injury. Laryngoscope.

[42] Feng X, Yuan W (2015). Dexamethasone enhanced functional recovery after sciatic nerve crush injury in rats. Biomed Res Int.

[43] Mohammadi R, Azad-Tirgan M, Amini K (2013). Dexamethasone topically accelerates peripheral nerve repair and target organ reinnervation: a transected sciatic nerve model in rat. Injury.

[44] Lundborg G, Dahlin LB, Danielsen N, Gelberman RH, Longo FM, Powell HC, Varon S (1982). Nerve regeneration in silicone chambers: Influence of gap length and of distal stump components. Experimental Neurology.

[45] Ghergherehchi CL, Bittner GD, Hastings RL, Mikesh M, Riley DC, Trevino RC, Schallert T, Thayer WP, Bhupanapadu Sunkesula SR, Ha TN, Munoz N, Pyarali M, Bansal A, Poon AD, Mazal AT, Smith TA, Wong NS, Dunne PJ (2016). Effects of extracellular calcium and surgical techniques on restoration of axonal continuity by polyethylene glycol fusion following complete cut or crush severance of rat sciatic nerves. J Neurosci Res.

[46] Riley D, Bittner G, Mikesh M, Cardwell N, Pollins A, Ghergherehchi C, Bhupanapadu Sunkesula S, Ha T, Hall B, Poon A, Pyarali M, Boyer R, Mazal A, Munoz N, Trevino R, Schallert T, Thayer W (2015). Polyethylene glycol-fused allografts produce rapid behavioral recovery after ablation of sciatic nerve segments. J Neurosci Res.

[47] Bittner G, Keating C, Kane J, Britt J, Spaeth C, Fan J, Zuzek A, Wilcott R, Thayer W, Winograd J, Gonzalez-Lima F, Schallert T (2012). Rapid, effective, and long-lasting behavioral recovery produced by microsutures, methylene blue, and polyethylene glycol after completely cutting rat sciatic nerves. J Neurosci Res.

[48] Green D, Wolfe S, Birch R, Quick T (2005). Chapter 30. Green's Operative Hand Surgery. Vol 2. 7th ed.

